# Comparing Generic Drug Markets in Europe and the United States: Prices, Volumes, and Spending

**DOI:** 10.1111/1468-0009.12279

**Published:** 2017-09-12

**Authors:** OLIVIER J. WOUTERS, PANOS G. KANAVOS, MARTIN McKEE

**Affiliations:** ^1^ London School of Economics and Political Science; ^2^ London School of Hygiene and Tropical Medicine

**Keywords:** generic drugs, health expenditures, pharmaceutical policies, prices

## Abstract

**Policy Points:**

- Our study indicates that there are opportunities for cost savings in generic drug markets in Europe and the United States.
- Regulators should make it easier for generic drugs to reach the market.
- Regulators and payers should apply measures to stimulate price competition among generic drugmakers and to increase generic drug use.
- To meaningfully evaluate policy options, it is important to analyze historical context and understand why similar initiatives failed previously.

**Context:**

Rising drug prices are putting pressure on health care budgets. Policymakers are assessing how they can save money through generic drugs.

**Methods:**

We compared generic drug prices and market shares in 13 European countries, using data from 2013, to assess the amount of variation that exists between countries. To place these results in context, we reviewed evidence from recent studies on the prices and use of generics in Europe and the United States. We also surveyed peer‐reviewed studies, gray literature, and books published since 2000 to (1) outline existing generic drug policies in European countries and the United States; (2) identify ways to increase generic drug use and to promote price competition among generic drug companies; and (3) explore barriers to implementing reform of generic drug policies, using a historical example from the United States as a case study.

**Findings:**

The prices and market shares of generics vary widely across Europe. For example, prices charged by manufacturers in Switzerland are, on average, more than 2.5 times those in Germany and more than 6 times those in the United Kingdom, based on the results of a commonly used price index. The proportion of prescriptions filled with generics ranges from 17% in Switzerland to 83% in the United Kingdom. By comparison, the United States has historically had low generic drug prices and high rates of generic drug use (84% in 2013), but has in recent years experienced sharp price increases for some off‐patent products. There are policy solutions to address issues in Europe and the United States, such as streamlining the generic drug approval process and requiring generic prescribing and substitution where such policies are not yet in place. The history of substitution laws in the United States provides insights into the economic, political, and cultural issues influencing the adoption of generic drug policies.

**Conclusions:**

Governments should apply coherent supply‐ and demand‐side policies in generic drug markets. An immediate priority is to convince more physicians, pharmacists, and patients that generic drugs are bioequivalent to branded products. Special‐interest groups continue to obstruct reform in Europe and the United States.

Rising drug prices are putting pressure on health care budgets.^1^, ^2^ Drugs account for sizable shares of health care spending in rich countries, with costs of new treatments for diabetes,^3^ multiple sclerosis,^4^ rheumatoid arthritis,^5^ various cancers,^6^, ^7^, ^8^, ^9^ and dermatological conditions^10^ increasing. There are many reasons, including aggressive pricing strategies by manufacturers^6^ and adoption of greater numbers of orphan and personalized drugs with high price tags.^11^, ^12^ Governments are responding by looking at ways to negotiate lower prices for patented drugs^13^ and to expand the use of health technology assessments to ensure medicines are given to those who will benefit most.^14^ Policymakers are also assessing how they can save money through generics.

Generic drugs are bioequivalent replicas of brand‐name drugs, containing the same active ingredients and with identical quality, safety, and efficacy profiles.^15^, ^16^, ^17^, ^18^ Any differences are limited to inactive ingredients, like coloring, flavoring, and stabilizing agents. Generics can, in theory, be sold for a fraction of the price of brand name drugs for 2 reasons. First, it is relatively cheap to bring a bioequivalent product to market. Second, the market for the drug typically already exists, significantly reducing marketing expenses.^19^

The cost‐saving potential of greater generic drug use makes it an attractive option for policymakers, especially since many blockbuster drugs went off patent in the last decade, with more soon to follow. Notably, the cholesterol‐lowering drug rosuvastatin (Crestor)—one of the best‐selling medicines of all time—lost market exclusivity in the United States and many European countries in 2016.^20^

We have 4 objectives. First, we compare generic drug prices and market shares in 13 European countries, using data from 2013, to assess the amount of variation that exists between countries. To place these findings in context, we review recent studies on prices and use of generic drugs in Europe and the United States. Second, we outline existing generic drug policies in European countries and the United States. Third, given issues identified in the earlier parts, we explore possible measures to increase usage of generics and to stimulate price competition among generic drugmakers. And, fourth, we analyze obstacles to improving generic drug policies, using a historical example from the United States as a case study.

## Methods

### Data Set

We selected 13 European countries with different generic drug policies: Germany, France, the United Kingdom, Spain, Italy, Poland, Switzerland, the Netherlands, Greece, Portugal, Belgium, Sweden, and Denmark (listed in order of pharmaceutical market size). For each country, we obtained IMS Health data on the 2013 sales of 200 off‐patent active ingredients (Appendix 1), available in 3,156 strength‐form combinations. These were the most‐prescribed off‐patent active ingredients in the European Union (EU) that year, according to IMS Health data. Sales were recorded in terms of volume and monetary value.

Volumes were measured in number of doses, which IMS Health sometimes refers to as “standard units.” IMS Health defined the amount in a single dose of each product, which could be 1 tablet, 5 mL of liquid, 1 vial, and so forth.^21^ We excluded 129 products (4.1%, 129/3,156) for which there was no information on dosage.

Monetary values were measured in euros, with foreign currencies converted to euros at yearly average exchange rates.^22^ These figures were obtained by multiplying the price of a product, excluding value‐added taxes, by the number of packs sold over the year. This was done using ex‐manufacturer and retail prices separately. Ex‐manufacturer prices were those charged by manufacturers to wholesalers, while retail prices were those charged by pharmacists to patients or insurers. Appendix 2 includes further details on the calculations.

The data set lacked certain information. First, it excluded biosimilar products, parallel‐traded generic drugs, off‐patent brand‐name drugs, and generics sold in hospital pharmacies. Second, retail data were unavailable for the Netherlands and the United Kingdom. Finally, the sales data did not reflect confidential rebates and discounts.

### Price Indexes

We calculated Laspeyres indexes to compare drug prices in 3 steps.^23^, ^24^, ^25^, ^26^, ^27^, ^28^, ^29^ First, for each active ingredient, we calculated the average price per dose by dividing the total sales across form‐strength combinations by the number of doses sold. For instance, omeprazole (Prilosec) was sold in France as 10‐mg and 20‐mg capsules. The ex‐manufacturer sales of these drugs amounted to roughly €88.5 million and 450 million doses. Accordingly, the average price per dose of omeprazole was €0.197 (88.5/450). We calculated the ex‐manufacturer and retail prices of each active ingredient.

Second, we identified a subset of 80 active ingredients prescribed in all 13 countries. This common sample accounted for between 46% and 72% of total generic drug sales in every country but the United Kingdom (25%). Table 1 shows descriptive statistics on the generic drug markets.

**Table 1:** Descriptive Statistics on Generic Drug Markets (2013)^a^

	Population (Millions)^b^	Generic Spending (Billions)^c^	Generic Spending (per Capita)^c^	Generic Volume (Billions of Doses)^c^	Generic Volume (per Capita)^c^	Proportion of Generic Spend Accounted for by the Sample^c^	Generic Market Share (Volume)^d^	Generic Market Share (Value)^d^
Belgium	11.2	€0.45	€40.6	4.2	251.6	56%	32%	14%
Denmark	5.6	€0.17	€29.6	2.7	481.6	56%	54%	14%
France	66.0	€4.14	€62.8	25.6	387.9	52%	30%	16%
Germany	82.1	€5.20	€63.4	37.6	458.3	51%	80%	37%
Greece	11.0	€0.45	€41.0	2.3	207.4	67%	20%	15%
Italy	60.2	€2.08	€34.5	15.3	254.0	47%	19%	11%
Netherlands	16.8	€0.50	€29.8	7.5	445.7	47%	70%	16%
Poland	38.0	€1.55	€40.9	16.2	425.5	46%	57%	42%
Portugal	10.5	€0.47	€45.1	2.8	401.1	49%	39%	23%
Spain	46.6	€2.12	€45.6	19.4	416.0	54%	47%	21%
Sweden	9.6	€0.32	€33.8	3.8	399.2	72%	44%	15%
Switzerland	8.1	€0.51	€63.4	1.8	231.7	71%	17%	16%
United Kingdom	64.1	€2.87	€44.8	36.3	566.0	25%	83%	33%

^a^All monetary figures are based on ex‐manufacturer prices. The market shares account for reimbursed generics in hospital and retail pharmacies.^30^

^b^Reproduced from the World Bank.^31^

^c^Reproduced from IMS Health (2013, Pricing Insights database).

^d^Reproduced from the Organisation for Economic Co‐operation and Development,^32^ with the exception of the Polish and Swedish figures (IMS Health, 2013).

Third, we calculated Laspeyres indexes using weights from a base country, in this case Germany, since it is the largest drug market in Europe by revenue. The rationale behind weighted indexes is that prices of highly consumed active ingredients should be given greater consideration. The indexes are calculated as:

$$I_{L}=\frac{\sum_{i=1}^{n}p_{i}^{c}q_{i}^{b}}{\sum_{i=1}^{n}p_{i}^{b}q_{i}^{b}}\;\cdot 100\;$$

where *p* is the price of an ingredient (*i*) in a comparator country (*c*) or the base country (*b*), and *q* is the corresponding quantity in doses. The base country is assigned a value of 100.

The Laspeyres results are interpreted as price ratios. For instance, an index value of 140 for *country X* means that prices are, on average, 40% higher there than in the base country (Germany). Conversely, a value of 60 for *country X* indicates that prices are, on average, 40% lower in *country X*. Because we limited our analysis to medicines available in all 13 countries, the indexes show how prices differ between each country. In other words, if values of 140 and 80 are observed for *countries X* and *Y*, respectively, it indicates that prices are, on average, 75% higher in *country X* than in *country Y* (140/80).

### Policy Analysis

To place the price‐index results in context, we first summarized evidence from recent studies on the prices and use of generic drugs in Europe and the United States. We then surveyed peer‐reviewed studies, gray literature, and books published since 2000 to (1) describe current generic drug policies in Europe and the United States; (2) identify potential solutions to increase generic drug use and to spur competition among generic manufacturers; and (3) explore barriers to the introduction of generic drug policies, using the history of substitution laws and bioequivalence regulation in the United States as a case study.

## Results

### Generic Drug Market Shares and Prices in Europe and the United States

Table 1 shows the proportion of prescriptions filled with generics in 13 European countries. The percentages were low (ie, less than 40%) in Switzerland (17%), Italy (19%), Greece (20%), France (30%), Belgium (32%), and Portugal (39%). They were moderate (ie, 40% to 60%) in Sweden (44%), Spain (47%), Denmark (54%), and Poland (57%); and high (ie, greater than 60%) in the Netherlands (70%), Germany (80%), and the United Kingdom (83%).

#### Price Indexes

**Figure 1:**
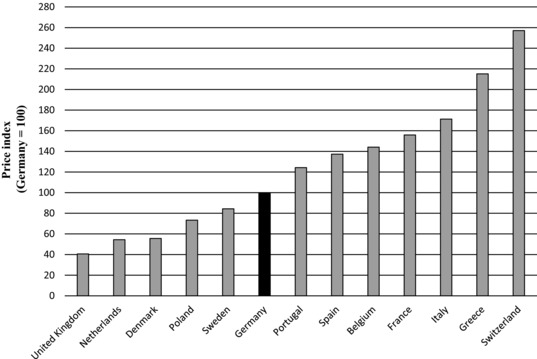
Ex‐Manufacturer Prices of Generics (2013)^a^ ^a^Derived from authors’ analysis of data from the Pricing Insights database (IMS Health, 2013).

Figure 1 compares ex‐manufacturer prices in each country. The figure shows wide variation in prices. For example, Swiss ex‐manufacturer prices were, on average, more than 2.5 times German ones and more than 6 times British ones.

**Figure 2:**
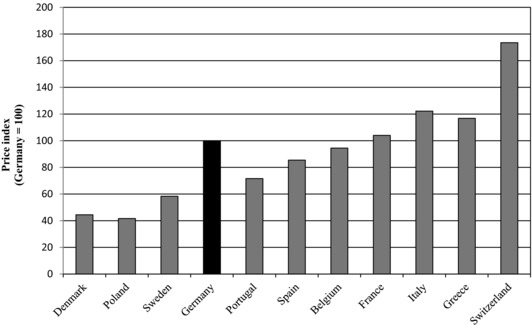
Retail Prices of Generics (2013)^a,b^ ^a^Retail prices were unavailable for the Netherlands and the United Kingdom. ^b^Derived from authors’ analysis of data from the Pricing Insights database (IMS Health, 2013).

Figure 2 compares retail prices in 11 European countries. The spread between the Swiss and German retail prices was smaller than the difference between the ex‐manufacturer prices. Retail prices in Portugal, Spain, and Belgium were lower than in Germany, whereas the opposite was true at the ex‐manufacturer level. Retail prices include distribution costs (ie, transport, processing, and storage) and markups charged by wholesalers and pharmacies.

Table 2 shows the ex‐manufacturer prices of 7 of the most consumed products in the sample. Atorvastatin (Lipitor) and simvastatin (Zocor) are cholesterol‐reducing drugs; amlodipine (Norvasc) is a calcium channel blocker used to treat high blood pressure and coronary heart disease; metformin (Glucophage) is a type 2 diabetes medication; and esomeprazole (Nexium), omeprazole (Prilosec), and pantoprazole (Protonix) are proton‐pump inhibitors used to treat heartburn and related conditions. Prices of all 7 products differ among countries. For instance, the price per dose of omeprazole was 30 times greater in Switzerland than in the United Kingdom (€0.811 vs €0.027). Even after excluding Greece and Switzerland, the 2 countries that generally had the highest prices, there were large price discrepancies.

**Table 2:** Ex‐Manufacturer Prices (€ per dose) of 7 Top‐Selling Active Ingredients (2013)^a^

	Amlodipine	Atorvastatin	Esomeprazole	Metformin	Omeprazole	Pantoprazole	Simvastatin
Belgium	€0.11	€0.20	€0.19	€0.03	€0.24	€0.20	€0.12
Denmark	€0.01	€0.12	€0.27	€0.01	€0.04	€0.03	€0.02
France	€0.14	€0.27	€0.19	€0.06	€0.20	€0.19	€0.19
Germany	€0.01	€0.07	€0.16	€0.02	€0.12	€0.17	€0.08
Greece	€0.17	€0.52	€0.27	€0.04	€0.45	€0.40	€0.40
Italy	€0.09	€0.13	€0.26	€0.03	€0.21	€0.22	€0.11
Netherlands	€0.02	€0.09	€0.11	€0.02	€0.03	€0.04	€0.02
Poland	€0.06	€0.12	€0.15	€0.04	€0.17	€0.09	€0.10
Portugal	€0.07	€0.12	€0.17	€0.04	€0.09	€0.09	€0.08
Spain	€0.04	€0.29	€0.43	€0.02	€0.06	€0.32	€0.04
Sweden	€0.02	€0.10	€0.18	€0.03	€0.11	€0.15	€0.05
Switzerland	€0.32	€0.40	€0.47	€0.05	€0.81	€0.30	€0.48
United Kingdom	€0.01	€0.03	€0.14	€0.02	€0.03	€0.03	€0.02
% difference (highest/lowest)	2,723%	1,990%	450%	469%	3,027%	1,492%	2,382%

^a^Derived from authors’ analysis of data from the Pricing Insights database (IMS Health, 2013).

Small price differences can have a large budget impact for high‐volume drugs. For example, roughly 294 million doses of simvastatin were consumed in France in 2013. For simvastatin alone, if France had paid the UK price per dose (€0.020 instead of €0.192), spending would have been more than €50 million less. There are caveats: volumes might not remain constant if prices change, and there might be differences in production and supply‐chain costs that prevent price equalization across countries.

#### Recent Evidence

Recent studies indicate there are opportunities for cost savings in off‐patent drug markets in Europe and the United States.

A high‐profile inquiry by the European Commission into generic competition found that patients in EU countries have to wait an average of about 7 months for generics to become available, starting from when brand‐name drugs lose market exclusivity.^33^ The inquiry report, published in 2009, estimated that these delays cost payers in EU countries €3 billion ($3.4 billion) per year, based on retail prices.^33^ Those findings were echoed by a 2014 study, which found significant delays in the availability of generics in many European countries.^34^

The European Commission's report showed that generics are slow to penetrate markets: after 2 years on the market, generics account for less than half of sales in EU member states.^33^ The report also found that prices are slow to drop in many countries. Variation in prices and market shares between European countries has been attributed to differences in pricing and reimbursement regulations, prescribing policies, and generic substitution laws, among other factors.^27^, ^33^, ^35^

By comparison, the United States has historically had high rates of generic drug use—84% of prescriptions were filled with generics in 2013^32^—and low prices.^2^ In recent years, however, it has seen a decrease in competition in the generics sector. Between 2012 and 2013, the total cost of 280 widely used generic medicines only fell by 4% in the United States, a slower rate of decline than in the previous 7 years.^36^ This trend was due to a combination of issues, including supply‐chain disruptions, loopholes in regulations by the US Food and Drug Administration (FDA), tough market conditions driving firms out of business, a flurry of mergers and acquisitions, and backlogs in the processing of generic drug applications by the FDA.^37^, ^38^, ^39^

In extreme cases, reduced competition has enabled individual companies to drastically raise the prices of generic drugs.^40^ For example, the price of pyrimethamine (Daraprim), an off‐patent anti‐infective medication, went up by about 5,500% overnight in 2015.^41^, ^42^ Such price hikes have affected numerous generic drugs, including the widely used antibiotic doxycycline (Doryx) and the cholesterol‐lowering drug pravastatin (Pravachol). The cost of 500 doxycycline capsules rose from $20 in October 2013 to $1,928 in April 2014, while the cost of a 1‐year supply of pravastatin rose from $27 to $196 during the same period, according to an analysis by the senior citizen group AARP.^43^ The US Government Accountability Office reported that between 2010 and 2015 there were “extraordinary price increases” of 100% or more for 315 out of the 1,441 generics they studied.^44^ Many of the affected medicines have been around for decades at low cost.^2^, ^39^, ^44^ (There have also been documented cases of large price hikes for generic drugs in some European countries, like the United Kingdom.^45^)

Moreover, recent studies show that many American and European physicians, pharmacists, and patients do not perceive brand‐name and generic drugs to be bioequivalent.^46^, ^47^, ^48^, ^49^, ^50^, ^51^, ^52^, ^53^, ^54^, ^55^, ^56^ A 2016 study found that 30% of surveyed physicians in the United States preferred prescribing brand‐name drugs over their generic counterparts, while 27% believed generics cause more adverse effects than brand‐name drugs.^50^ A 2013 US study reported that 2 in 5 physicians “sometimes” or “often” prescribe brand‐name drugs instead of equivalent generics when patients request the former.^57^

In summary, there are shortcomings in generic drug markets in Europe and the United States, notably delays in the availability of generics, high prices, and low utilization rates. These issues affect countries to varying degrees. In the next section, we outline contemporary generic drug policies in Europe and the United States to identify lessons that might be drawn from different approaches.

### Generic Drug Policies in Europe and the United States

There are vast differences between countries in terms of regulatory structures, lobbying powers of special‐interest groups, patent‐litigation systems, political economies of health care systems, and perceptions of generics among patients and health care professionals.^58^ Such differences influence the adoption and effectiveness of policies.

**Figure 3:**
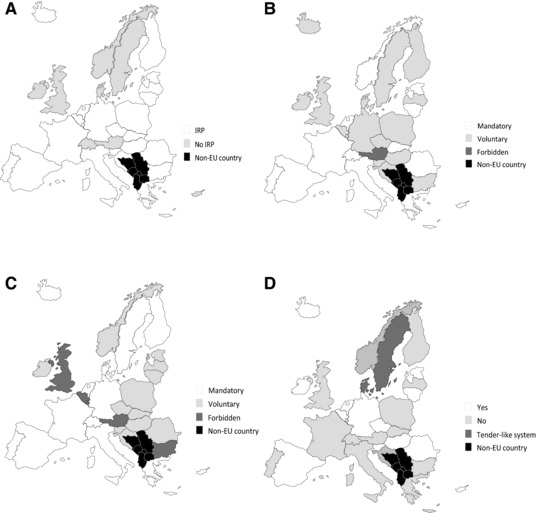
Internal Reference Pricing (A), Generic Prescribing (B), Generic Substitution (C), and Tendering (D) in EU and EFTA Countries (2016)^a,b^ Abbreviations: EFTA, European Free Trade Association; EU, European Union; IRP, internal reference pricing. ^a^These maps show the policies used by the 28 EU member states and the 4 EFTA signatories (Iceland, Lichtenstein, Norway, and Switzerland) for nonhospital pharmacies. We populated the maps based on a 2016 report published by the World Health Organization.^64^ If information was missing, we used older sources dating as far back as 2009. The policies in some countries may have changed since then. In Spain, only the autonomous community of Andalusia issues tenders. Generic prescribing refers to the prescribing of drugs by their international nonproprietary names. The Danish and Swedish tendering systems operate differently than the others. In each country, the relevant national government agency asks generic manufacturers to offer their best prices. Usually, the least expensive generics become the only ones that pharmacists can dispense; if a patient wants a brand‐name drug, they are required to pay the difference out‐of‐pocket. The bidding process is repeated every 2 weeks in Denmark, and every 4 weeks in Sweden. There are safeguards to reduce the risk of supply disruptions. ^b^Derived from authors’ analysis of the data^62^, ^63^, ^64^, ^65^, ^66^, ^67^, ^68^; the map toolkit is licensed under the Creative Commons Attribution‐NoDerivs 3.0 Unported License.

Figure 3 shows the patchwork of policies in place in Europe. Generic drug substitution is mandatory in 13 countries, voluntary in 14, and forbidden in 5. The situation with respect to generic prescribing is similarly diverse. Internal reference pricing, which limits how much insurers will reimburse for generics, is used in most countries.^59^, ^60^, ^61^ In several countries, health insurers buy generic drugs in bulk from the manufacturers that offer the best prices, a policy referred to as tendering.^62^, ^63^ For example, a health insurer might put out a tender for 1 million packs of 20‐mg simvastatin and ask generic manufacturers to submit confidential bids. The winning manufacturer is asked to supply the entire market for the duration of the contract, which typically ranges from 1 to 2 years.^63^

Figure 3 gives a broad overview of policies, but the way these policies are implemented varies considerably. Other supportive measures are often used to influence generic drug usage, such as charging higher co‐payments on branded drugs that have generic equivalents to encourage patients to choose the latter.

National governments in all but 3 EU member states (Denmark, Germany, and the United Kingdom) impose price controls on generics (ie, maximum allowable prices).^65^ Often these controls are linked to the prices of brand‐name drugs. In Spain, for instance, the first company to sell a generic version of a drug must price its product at least 40% below the price of the brand‐name drug at the time of loss of market exclusivity; subsequent generic entrants must be priced at or below this level. Many EU governments also retain the right to block large price increases for prescription drugs, including generics, if necessary to protect public health or reduce pressure on the public purse.^64^ As nearly all EU countries have universal health care systems, funded either through government tax revenues or taxes on employers and employees, a population‐based focus has strong political support from consumers and nonindustry stakeholders in these nations.^69^

**Figure 4:**
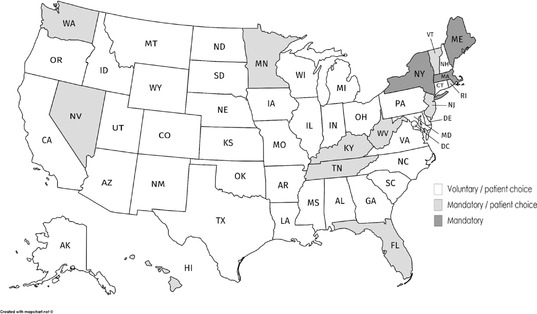
Generic Drug Substitution Laws in the United States (2010)^a,b^ ^a^States with “patient choice” grant patients the right to refuse generic drug substitution, usually at a higher cost. ^b^Derived from authors’ analysis of data^64^; the map toolkit is licensed under the Creative Commons Attribution‐ShareAlike 3.0 Unported License.

In the United States, by comparison, generic prescribing is voluntary in all 50 states. Neither internal reference pricing nor tendering is used for generic drugs sold in nonhospital pharmacies. There are no government price controls on generics, and substitution laws differ from state to state, as shown in Figure 4.^70^

**Figure 5:**
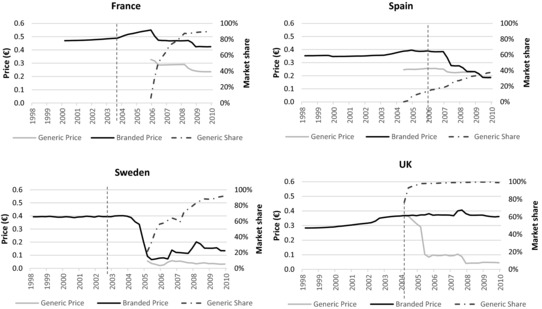
Ex‐Manufacturer Prices (€ per Dose) and Market Shares (%) of Brand Name and Generic Ramipril in 4 Countries (1998‐2010)^a,b^ ^a^The vertical lines show the date of patent expiry (November 2002 in France, Sweden, and the United Kingdom; March 2003 in Spain). The Swedish and British prices were converted to € using official exchange rates. The data correspond to sales in nonhospital pharmacies (except for Sweden, where the data include sales in hospital and nonhospital pharmacies). Data on sales of branded ramipril in France between January 1998 and December 1999 were unavailable. Prices and market shares were measured on a quarterly basis. ^b^Derived from authors’ analysis of data from the Midas database (IMS Health, 2010).

Pricing, prescribing, and substitution policies can affect the prices and usage of generics.^33^ To illustrate this, Figure 5 shows how ex‐manufacturer prices and market shares of ramipril (Altace), a drug widely used to treat high blood pressure, evolved between 1998 and 2010 in 4 countries. Ramipril lost patent protection in each country in either November 2002 or March 2003, as indicated by the vertical lines.

Figure 5 shows that it took over a year for the first generic version of ramipril to come on the market in the United Kingdom and Spain, compared to 2 years in Sweden and 3 years in France. The trends in prices and market shares in each country varied considerably. In the United Kingdom, the generic price fell to about a fifth of the branded price within 3 months of the first generic being launched. During this time, the price of the branded version remained unchanged and generic ramipril captured over 90% of the British market. In Spain, on the other hand, the generic competitor was introduced at about 60% of the branded price and only slowly gained market share, reaching around 10% after 1 year of being on the market and only 25% after 3 years. The branded price then fell to match the generic, which showed no sign of responding to competition. In Sweden, at launch, the generic price was only 10% of the branded price, which rapidly fell to a similar level. The generic market share continued to rise steeply, to almost 100%. In France, although the prices moved in step, the price of the generic drug remained about two‐thirds that of the branded. Again, the generic gained a high market share within a few years of entering the market. By the end of 2009, generic ramipril cost 7 times more in France (€0.236 per dose) than in Sweden (€0.033).

### Possible Policy Solutions

Having summarized the policies adopted in various countries, we now outline measures that appear to be effective in promoting price competition among generic drug companies and increasing the use of generic drugs, based on available evidence. These are generally applicable to both European countries and the United States, wherever such policies are not yet in place, although decisions on which ones to implement in individual countries require detailed market analyses.

#### Facilitate Generic Market Entry

First, national regulators should streamline the generic drug approval process. In response to recent price hikes in the United States, Kesselheim and colleagues called for regulators to prioritize applications from manufacturers trying to bring to market a generic medicine sold by 3 or fewer firms.^2^, ^37^ This would put downward pressure on prices and make it harder for individual companies to have much influence over prices. For off‐patent drugs facing limited or no competition, Kesselheim and colleagues further recommended that the FDA temporarily import generics from countries with equally high regulatory standards, like Canada and EU member states, to avoid paying high premiums.^2^, ^71^

Second, in countries with backlogs of applications for generic drug approval, governments could allocate more resources to national regulators to speed up the review process^2^ or could charge generic firms fees to increase resources available for the drug approval process, as is done by the FDA.^72^ In the European Union, levels of backlogs vary greatly between national regulatory agencies, despite efforts to harmonize such processes across the union.^31^ In the United States, it currently takes about 15 months, on average, for generic drugmakers to receive an initial response from the FDA.^73^ Over 4,000 generic drugs were awaiting approval from the FDA as of mid‐2016.^74^

Third, regulators should address the anticompetitive tactics used by brand‐name firms to delay generic drug launches. Brand‐name manufacturers frequently file patent infringement lawsuits against generic drugmakers for launching their drugs too early, preventing the marketing of generic products while the companies are tied up in court.^33^ Some such lawsuits might reflect calculations by brand‐name firms that the extra revenue obtained after patent expiry is likely to exceed the legal fees incurred.^75^ Brand‐name firms have employed other strategies to hinder market entry for generic drugs, like filing patent clusters (ie, complex webs of primary and secondary patents on pharmaceutical products and manufacturing processes that serve to extend periods of market exclusivity).^33^, ^76^, ^77^ Some medicines are protected by as many as 1,300 patents,^33^ making it difficult for generic drugmakers to determine when they can legally enter the market. The US Congress proposed new legislation in 2015 that could make it easier for generic drug companies to challenge patents without the need for lengthy and costly litigation.^78^ The bill is still under consideration. The European Commission has called for similar measures.^33^

Fourth, regulators should block pay‐for‐delay deals, where brand‐name drugmakers offer generic manufacturers cash, or something else of value, to delay the introduction of generic drugs onto the market.^33^ Brand‐name drugmakers continue to enjoy monopolies, meaning consumers pay higher prices for longer. These deals happen in both Europe and the United States. In 2009, the US Federal Trade Commission (FTC) estimated that eliminating pay‐for‐delay deals would save consumers and the federal government over $3.5 billion a year.^79^ A 2013 Supreme Court ruling gave the FTC the authority to block such deals, which it has begun to do. Yet there continue to be legal disputes over what constitutes a pay‐for‐delay deal, which hamper the FTC's efforts.

Finally, regulators should facilitate access to samples of brand‐name products for generic drugmakers. Since 2007, some brand‐name manufacturers have taken advantage of a legal loophole in the United States to block access to samples, citing restrictions imposed by the FDA through risk evaluation and mitigation strategies.^80^, ^81^ This prevents generic drug companies from conducting bioequivalence tests prior to patent expiry. These test results are needed for companies to receive marketing authorization at the time of patent expiry. Several countermeasures have been proposed by Congress and the FDA, but none have been implemented to date.^80^

#### Encourage Price Competition

Studies on pricing policies indicate that allowing generic drug companies to set their own prices, while giving physicians and pharmacists incentives to prescribe and dispense the least expensive generics, is more effective at driving down prices over time than government‐mandated price controls.^26^, ^33^, ^82^, ^83^, ^84^, ^85^ This is the approach adopted by policymakers in Denmark, Sweden, the United Kingdom, and the United States,^35^ although the Swedish authorities reserve the right to block large price increases for generics. An analysis of IMS Health data conducted by the FDA found that drug prices in the United States drop, on average, by around 50% with 2 generic competitors on the market, around 70% with 5 on the market, and around 90% with 15 or more on the market.^86^ However, safeguards are needed to prevent large, unjustified price hikes for drugs available in generic versions. For example, increases exceeding a percentage threshold could be blocked by national authorities on economic or public health grounds, with exceptions for causes outside the control of manufacturers which are verifiable (eg, changes in the prices of ingredients).

Tendering is another way to encourage price competition, especially if market competition fails to achieve large price reductions for generic medicines. As mentioned earlier, tendering refers to the purchase of generics in bulk, usually from the suppliers offering the lowest prices. It has been shown to lower administrative costs, drive down the prices of generics, and improve price transparency.^62^, ^63^, ^87^, ^88^ In the Netherlands, for example, the introduction of tendering resulted in the retail prices of some generics—including amlodipine, omeprazole, and simvastatin (see Table 2)—dropping by 80% to 90% overnight in nonhospital pharmacies.^87^ The color, shape, and size of a pill might change after a tender if a new manufacturer is asked to supply the market, so physicians and pharmacists need to communicate such changes to patients to promote treatment adherence.^89^, ^90^, ^91^ Also, European payers in charge of tendering sometimes split contracts between 2 or more manufacturers, as long as the bids are close to each other, to minimize the risk of supply disruptions and to maintain competition.^63^ There is no conclusive evidence, though, that disruptions occur more often in countries that rely on tendering than in others.

#### Promote Generic Dispensing and Prescribing

Countries should require pharmacists to substitute generic drugs for brand‐name medicines. The Swedish national government, for example, introduced mandatory generic substitution in 2002, which led to a spike in generic drug use.^92^ The European Commission found that generic drugs enter the market sooner, on average, in EU member states with mandatory substitution.^33^ Currently, generic substitution is mandatory in only 11 EU countries and 14 US states.^64^, ^70^

Governments should encourage or require physicians to prescribe drugs by their generic names.^35^, ^93^ A recent study estimated that physicians blocking generic drug substitution costs the United States over $7.5 billion per year, including $1.2 billion in out‐of‐pocket fees for patients.^94^ This practice is also costly in European countries, including France^95^ and Switzerland.^96^ There may be legitimate reasons for prescribing brand‐name drugs instead of generic ones—for example, a patient might be allergic to an inactive ingredient in a generic medicine.^89^ However, in many cases, those decisions are likely due to habit or misconceptions about generic medicines among physicians.^70^ Academic detailing (ie, having trained experts with no conflicts of interest provide unbiased information to clinicians about the effectiveness, safety, and costs of drugs) could help correct suboptimal prescribing.^97^ A meta‐analysis conducted for Cochrane found, based on data from 25 randomized controlled trials, that academic detailing improves compliance with desired prescribing practices.^97^ Financial incentives aimed at improving rates of generic prescribing were also shown to be effective, although the evidence base is limited.^98^, ^99^

Moreover, regulators in some countries allow pharmacists to substitute a generic for a brand name drug with a different active ingredient, as long as both drugs belong to the same therapeutic class and have the same indication. For example, if a doctor prescribes a patient rosuvastatin, a cholesterol‐lowering drug not yet available in generic form in some countries, a pharmacist could give the patient generic simvastatin instead.^100^ A recent study estimated that the United States spends an extra $73.0 billion per year—about 10% of total drug spending—on brand‐name drugs with available therapeutic substitutes. This estimate included $24.6 billion in out‐of‐pocket expenses.^101^ Most of the estimated excess spending was on brand‐name drugs in 5 classes: statins, a class of cholesterol‐reducing drugs ($10.9 billion); atypical antipsychotics, a class of drugs used to treat psychiatric conditions ($9.99 billion); proton pump inhibitors, a class of drugs used to treat heartburn and related conditions ($6.12 billion); selective serotonin reuptake inhibitors, a class of drugs used to treat depression ($6.08 billion); and angiotensin receptor blockers, a class of drugs used to lower blood pressure ($5.53 billion).^101^

Therapeutic substitutes can vary in terms of side effects and other properties, so this form of substitution is less straightforward to implement than substitution of bioequivalent products. For therapeutic substitution to be more widely practiced, the relevant authorities and clinical organizations should develop appropriate protocols and strengthen coordination between physicians, pharmacists, and insurers.^101^, ^102^, ^103^ A challenge is to get buy‐in from trade groups for physicians, many of which have, in the past, opposed such restrictions on prescribing and have raised concerns about the potential adverse health consequences for patients.^101^, ^104^ In the United States, some patient organizations have also been skeptical of therapeutic substitution, worried that legislators are too focused on cutting costs at the expense of quality of care.^103^

### Barriers to Reforming Generic Drug Policies: A Case Study From the United States

Having reviewed a range of policy options, we now draw on the experience of one country, the United States, to explore barriers to reform and offer thoughts on how they might be overcome. While we focus on the history of substitution and bioequivalence policies in the United States as a case study, similar analyses could be done for any country.

**Figure 6:**
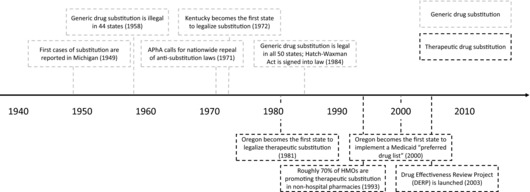
Key Events and Milestones for US Drug Substitution^a^ Abbreviations: APhA, American Pharmacists Association; HMO, health maintenance organization. ^a^Derived from authors’ analysis of the data.^103^, ^112^

The history of generic drug substitution in the United States (Box 1) shows how trade groups for brand‐name drugmakers and clinicians have consistently banded together to resist generic drug policy reform in the United States.^105^, ^106^, ^107^ It is a history marked by political conflicts, vested economic interests, and intense lobbying by stakeholders.^103^ Figure 6 highlights key events and milestones.

Box 1 **History of Drug Substitution in the United States^a^**

*Generic Drug Substitution*

The first instances of generic drug substitution were reported in the late 1940s. In response, the National Pharmaceutical Council (NPC), a trade organization for the brand‐name drug industry, began aggressively lobbying against substitution, saying it would stifle innovation. The group further claimed that substitution would reduce quality of care, citing the scientific uncertainty that existed at the time over whether generic drugs were as effective as brand‐name drugs.^108^

The NPC forged an alliance with the American Medical Association (AMA) and the American Pharmacists Association (APhA), 2 major trade groups for clinicians and pharmacists. The AMA argued that substitution diminished the role of physicians, while the APhA said it was a violation of the ethical and professional standards of the trade. (In an apparent quid pro quo, the NPC helped pharmacists lobby against supermarkets, which were beginning to sell prescription and over‐the‐counter drugs.) The AMA was further concerned that government intervention on dispensing was a step toward socialized medicine, which they opposed.

The anti‐substitution campaign was largely successful: by 1959, 44 states had enacted laws blocking generic drug substitution.

During the 1960s and ’70s, when state health care budgets were ballooning, state and municipal governments started looking at ways to cut health care spending. Meanwhile, there was growing support for substitution among pharmacists, who sought a more active role in the care of patients. In 1972, Kentucky became the first state to abolish its anti‐substitution law. By 1984, all 50 states had legalized generic drug substitution.

However, state policies differed in 3 ways. First, generic substitution was compulsory in some states and voluntary in others. Second, patients in many states could refuse substitution. Finally, some states restricted which drugs pharmacists could substitute.

The rollback of anti‐substitution laws on a state level resulted in a patchwork of policies, most of which remain in place today. Physicians in all states can block substitution, usually by ticking a box on the prescription pad that reads “dispense as written.”^70^ The poorer states have some of the weakest substitution laws in the country, leading one commentator to recently note that “the cost savings of generic substitution [in the United States] now appear to benefit populations in inverse proportion to economic need.”^103^

To date, all attempts by federal legislators to enforce a minimum standard of substitution have been voted down, and the politics and economics of substitution have continued to play out at the state level.^109^ Still, substitution laws have helped dramatically increase the rate of generic drug use in the United States: around 10% of prescriptions were filled generically in 1958, compared to 88% in 2015.

*Therapeutic Drug Substitution*

In the 1980s, state lawmakers and hospital administrators turned their attention to therapeutic substitution. Proponents argued that many new drugs offered little or no additional therapeutic benefit over existing ones and that they should be substituted for older, cheaper medicines—preferably generics. This would generate savings and incentivize drug companies to develop innovative products. Trade groups for brand‐name drugmakers and clinicians opposed therapeutic substitution, claiming it would harm patients.

Oregon passed the first therapeutic substitution law in 1981, and hospitals around the country began implementing a 2‐tiered approach: automatic therapeutic substitution in clear‐cut cases (eg, cephalosporins, anti‐allergy drugs, and heartburn treatments) and prior authorization in less straightforward cases (eg, beta‐blockers and anti‐cancer drugs). Between 1987 and 1993, the proportion of health maintenance organizations that allowed therapeutic substitution in nonhospital pharmacies doubled to 70%.

Private and public insurers increasingly turned to pharmacy benefit managers (PBMs), who serve as intermediaries between drug companies and payers, to help coordinate therapeutic substitution. PBMs negotiate lower drug prices and rebates on behalf of large patient populations. Most PBMs operate formularies specifying the preferred products for different therapeutic indications. These organizations help dictate the nature and extent of generic and therapeutic substitution. They often rely on tiered copayment systems, whereby patients are required to pay more for brand‐name drugs.

The lack of transparency with PBMs, however, meant that insurers were unsure about how much of the negotiated discounts was passed on to them, and how much was kept by PBMs. Some PBMs were bought by pharmaceutical companies, introducing further conflicts of interest.

In 2000, partly in response to the rapid growth and opaqueness of PBMs, the Oregon state legislature implemented guidelines on which medicines should be prescribed to Medicaid patients for specific conditions, known as a preferred drug list.^110^, ^111^ The preferred drug list was the “public, transparent, evidence‐based analogue of the private formulary‐shaping activities of the PBMs.”^103^ Idaho and Washington quickly followed suit and developed their own lists.

These 3 states joined forces with the Pacific Northwest Evidence‐based Practice Center in 2003 to form the Drug Effectiveness Review Project (DERP), a collaboration between Medicaid and public pharmacy programs in member states to promote evidence‐based prescribing. By 2008, the DERP consortium comprised 15 states and 2 nonprofit organizations, and 33 states operated preferred drug lists, most of which promoted therapeutic substitution wherever possible.

The economic downturn of 2008 put the project under financial strain. In 2014, there were only 9 paying members in the consortium. Still, DERP paved the way for future research into comparative effectiveness, a field fraught with ethical, political, methodological, organizational, and procedural issues.

^a^Derived from authors’ analysis of data^103^, ^105^, ^112^; other references are shown in the text.

Regulation of bioequivalence has played a key role in the evolution of substitution policies in the United States.^19^, ^103^, ^108^ In the 1950s and ’60s, when US lawmakers started calling for generic prescribing and substitution, there was little clarity about how to verify that generic drugs would produce the same therapeutic effects as their brand‐name counterparts. A scandal erupted in 1967 when it was found that some patients who consumed generic versions of chloramphenicol (Chloromycetin), a widely used antibiotic, had no traces of the active ingredient in their bloodstreams. It was later shown that the coating used by some generic manufacturers prevented the drug from dissolving in the gastrointestinal tract.^101^

In response, between 1967 and 1975, the US FDA commissioned 5 separate external committees to provide input on how to assess the therapeutic equivalence of generic and brand‐name drugs.^103^ The proliferation of committees and recommendations slowed down the market entry of generic drugs, hurt the public perception of generics, hampered the campaign to roll back anti‐substitution laws, and delayed other changes to generic drug policies during this period.^103^, ^105^, ^113^ The scientific and regulatory uncertainty around bioequivalence created space for brand‐name manufacturers and their trade groups to nurture brand loyalty and to claim, often without evidence, that there were meaningful differences between branded and generic medicines.^103^, ^113^ Not until 1984 did the FDA settle on a coherent and widely accepted set of bioequivalence standards—based on the rate and extent of absorption of the active ingredient into the bloodstream^17^—as part of the Drug Price Competition and Patent Term Restoration Act, more commonly known as the Hatch‐Waxman Act.^108^

The issues raised by the proponents and critics of generic drug policy reform have remained similar over the past 50 years in the United States.^105^, ^114^ The evolution of substitution and bioequivalence regulation provides insights into the economic, political, cultural, and scientific issues influencing policy changes. Such insights can help policymakers avoid past pitfalls.

#### Current Opportunities for Reform

Recent developments point to an opportunity for reform of generic drug policies in the United States.

In the past few years, a series of price scandals shifted public attention from the high prices of new medicines to the rising costs of generics, raising pressure on companies and policymakers to contain costs.^115^ A 2015 national survey by the Kaiser Family Foundation found that roughly 3 in 4 Americans believe prescription drug prices are unreasonably high, and, of those, 76% say pharmaceutical companies are mostly to blame.^116^ These findings may partly reflect the reputational damage to generic drugmakers caused by recent scandals, as well as the increase in the number of patients facing higher deductibles for medicines.^117^

A subsequent Kaiser poll, in 2016, found that the vast majority of Americans are in favor of government action to curb prescription drug prices.^118^ According to the results, more than 8 in 10 Americans (82%) favor allowing Medicare to negotiate prices with companies, while 66% support the creation of an independent group to oversee the pricing of prescription drugs and 71% believe patients should be allowed to buy medicines imported from Canada.^118^

The increasing roles of federal and state governments in health care has further renewed attention on cost containment.^103^, ^112^, ^119^ A growing number of government officials, including Senators Bernie Sanders (D‐VT), Susan Collins (R‐ME), Elijah Cummings (D‐MD), and Claire McCaskill (D‐MO), are looking at ways to improve competition in the off‐patent drug market to reduce spending, with some arguing that state and federal governments should be allowed to block unjustified price increases on generics.^120^, ^121^ Competition authorities are also investigating potential price collusion between generic companies.^121^, ^122^, ^123^ Private health insurers, which have a strong interest in keeping generic prices low, have joined the debate, arguing on the side of lawmakers on this issue. They were largely absent from discussions in the 1970s, ’80s, and ’90s when the prices of drugs were rising more slowly than those of other health care goods and services.^103^

The outcome of the 2016 presidential election could provide further momentum for improving generic drug policies. During the campaign, now‐President Donald Trump supported giving Medicare greater power to negotiate drug prices and allowing states to import less expensive drugs from Canada and elsewhere. He launched an attack on the pharmaceutical industry at a press conference a few days prior to his inauguration. “Pharma has a lot of lobbies, a lot of lobbyists, and a lot of power,” Trump said at the press conference. “And there's very little bidding on drugs. We're the largest buyer of drugs in the world, and yet we don't bid properly. And we're going to start bidding and we're going to save billions of dollars.” Since taking office, Trump has reiterated his support for Medicare drug price negotiations. He has also stated his desire to streamline the FDA drug approval process, but without offering specifics on how he would like to see the process for generic drugs changed.

Scott Gottlieb, Trump's new FDA commissioner who has close links to the pharmaceutical industry and a conservative think tank, also criticized the costs and delays of generic drug approvals. In his first remarks to FDA staff, Gottlieb urged the agency to take “meaningful steps to get more low‐cost alternatives to the market, to increase competition, and to give consumers more options.” He went on to say that the FDA should “make sure the generic drug process isn't being inappropriately gamed to delay competition and disadvantage consumers.”^124^ There are concerns, however, that changes to FDA procedures may harm the organization's ability to guarantee the efficacy, quality, and safety of approved drugs, including generics.^125^ While it remains to be seen how these developments play out in practice, the available evidence suggests a willingness by the Trump administration to address price hikes and to ensure the availability of low‐cost generics. It is critical, though, that any changes to FDA procedures do not undermine the agency's ability to ensure that approved generics, and new medicines, meet adequate regulatory standards.

Yet, at this writing, American health policy is extremely uncertain,^126^, ^127^, ^128^, ^129^, ^130^ and there are reasons why changes to generic drug policies may prove elusive.

In January 2017, the Republican‐controlled Congress approved a budget resolution that sets the stage for a major overhaul of the health care system, an action supported by Trump's health secretary.^129^ In May of this year, Republican lawmakers in the House of Representatives passed a bill to repeal and replace the Affordable Care Act. Among other things, the bill would eliminate tax penalties for Americans who do not have health insurance, remove a mandate for larger companies to offer affordable insurance to employees, increase annual limits on how much individuals and families can contribute to health savings accounts, cut taxes on high‐income individuals and other groups imposed by the Affordable Care Act, repeal income‐based tax credits and subsidies for out‐of‐pocket costs, remove caps on how much health insurers can charge older customers in monthly premiums, and cut federal funding for Medicaid, a publicly funded insurance program for low‐income individuals and families.^131^

An analysis conducted by the nonpartisan US Congressional Budget Office (CBO) estimated that the House bill would save the federal government over $100 billion in a decade, but would also drastically increase the number of uninsured over the next 10 years and lead to hikes in health care premiums in the coming 3 years.^132^ In many states, health care premiums and out‐of‐pocket costs would soar for chronically ill patients and decline for young and healthy individuals. It is unclear what impact such changes would have on the generic drug market, with the CBO report silent on this issue.

The bill moved to the Senate for a debate and vote, where Republican lawmakers proposed an amended version of the legislation, which included only modest changes. However, the Senate bill was defeated in July of this year following opposition from lawmakers on both sides of the aisle and key stakeholders, including the American Hospital Association and AARP. At the time of writing, Senate Republicans have indicated that they will postpone efforts to repeal and replace the Affordable Care Act, although Republican lawmakers can table new health care legislation at any point.

Moreover, the American Medical Association and the Pharmaceutical Research and Manufacturers of America, 2 of the largest and most influential lobbying organizations in the United States, continue to oppose government intervention in the generic drug market (see Box 1), such as stronger substitution laws and measures to block large price increases.^105^ Although both groups supported the Affordable Care Act, they did so only after having received assurances that there would be no price controls on medicines and no importation of cheaper medicines from other countries, among other conditions.^105^

## Discussion

A mix of factors, including aging populations, slowing economic growth, and rising costs of new drugs and medical technologies, have put pressure on governments to contain health care spending. Substituting generic medicines for more expensive brand‐name versions is likely among the most cost‐effective interventions in health care systems.^133^, ^134^, ^135^, ^136^, ^137^, ^138^, ^139^

Addressing issues in the generic drug sector can enhance equitable access to medicines in countries where patients face high out‐of‐pocket drug costs, like Cyprus,^140^ Greece,^141^ and the United States.^142^ Several studies indicate that patients who use generic medicines instead of brand name ones are more likely to adhere to treatment,^143^, ^144^ probably because of greater affordability, which can improve health outcomes.^143^

Between 2008 and 2015, in the wake of the global economic recession, several European governments implemented generic drug policies to help control costs.^64^, ^145^, ^146^, ^147^ During this period, Slovakia introduced voluntary generic prescribing, which was previously forbidden. Belgium, Estonia, Greece, Portugal, and Spain made generic prescribing compulsory. Greece and Portugal made generic substitution compulsory. And Finland introduced internal reference pricing.

Yet, as our results show, there remain large differences in the usage and prices of generics in Europe and the United States. The barriers to market entry for generic companies vary between countries, as do pricing and reimbursement policies. Beyond such features of the market, there are differences in whether, and to what extent, patients and health care professionals perceive generic and branded medicines to be bioequivalent.^46^, ^47^, ^54^ In some countries, negative perceptions of generics may have contributed to slower uptake of stronger prescribing and substitution measures.

Governments should apply coherent supply‐ and demand‐side policies in generic drug markets.^148^ There are interesting examples from smaller European countries, like Denmark,^149^ Norway,^150^ and Sweden,^149^ which have achieved low generic drug prices. There is no one‐size‐fits‐all solution, though, and there are different ways of achieving similar results. For instance, the United Kingdom is one of the few EU countries to forbid generic substitution. The electronic prescribing system in the United Kingdom, however, automatically prompts physicians to prescribe generic drugs when available. The country has one of the highest rates of generic drug use in the world,^151^ although some analysts argue that substitution should still be made mandatory in the United Kingdom since physicians can be influenced by the marketing of drug companies.^152^

The appropriate steps to reduce generic drug prices and to boost demand for such medicines will vary between countries. For example, in nations with historically high rates of generic drug use and low generic drug prices, but which are experiencing generic drug shortages, like the United States, the emphasis should be on facilitating market entry for generic drug companies. In countries with low rates of generic drug use, like Greece and Italy, more should be done to improve the perceptions of generics among physicians, pharmacists, and patients.

Finally, it is important to trace the cultural, political, regulatory, and scientific issues influencing the adoption of generic drug policies. Historical analyses can help policymakers avoid past stumbling blocks when trying to enact reform.^153^ For example, in a comparative study of drug regulation in the United States and Germany, Arthur A. Daemmrich analyzed the evolution of the medical and political settings of each country during the 20^th^ century, highlighting points of convergence and divergence.^58^ Daemmrich noted that legislative changes to prescription‐drug laws in the United States often occur in response to public scandals. In Germany, by contrast, changes tend to follow protracted negotiations between lawmakers and stakeholders.^58^ Drug regulation is highly politicized and adversarial in the United States, but much less so in Germany, where health care is widely seen as a right.^58^ Such political and cultural factors help to explain differences in generic drug policies among countries. Moreover, pharmaceutical policies involve balancing the interests of the health care system with those of the pharmaceutical industry, with this balance varying among countries.

### Limitations

The price comparisons in this study have limitations. First, an assumption behind the Laspeyres index is that demand for prescription generic drugs is price inelastic (ie, change in the price of a generic does not affect demand). Although empirical data suggest that this is unlikely to be true,^154^, ^155^ other types of weighted indexes make assumptions that might be less likely to hold.^24^, ^25^, ^26^ Laspeyres indexes are therefore commonly used to compare drug prices.^23^, ^24^, ^25^, ^149^, ^156^

Second, the IMS Health data do not reflect confidential rebates and discounts. The list prices (ie, official prices before discounts) may overestimate the actual prices paid for some products.^157^ Even so, list prices are meaningful to payers since they are the starting point for discount negotiations. It is important to strengthen price transparency in generic drug markets, since opaque pricing makes it easier for drugmakers to charge the highest prices markets will bear.

Third, to aggregate price data across drug forms and strengths, it is necessary to use a common unit of volume. As Danzon and Kim explained, “the ideal unit would be a quality‐constant … course of therapy for a given drug, which should be applicable to all [forms] and strengths. Such ideal units are not observable.”^24^ In calculating prices per dose, we implicitly assume that a single dose of a drug, in any form or strength, is of equal therapeutic value to all patients. Some studies have instead calculated prices per gram of active ingredient, but this measure suffers from other limitations.^24^

Finally, we had to exclude 4.1% of the drugs in our sample due to missing information on dosage. These were mostly aerosol, cream, gel, injectable, and powder products. This might have influenced our findings if there were systematic differences across countries in the prices of those types of products. Still, the common sample accounted for a large share of total generic sales in every country but the United Kingdom (25%). The UK results should be interpreted with caution.

### Conclusions

Greater use of generic medicines is one way to constrain growth in health care spending at a time when this is a political imperative everywhere. Yet, across high‐income countries, generic prices and market shares vary widely. This is despite the existence of effective policies to reduce delays in generic availability, stimulate price competition, and increase generic drug use. There are, however, signs of change. European payers and policymakers are showing growing interest in tendering to lower prices, something that seems to be effective.

An immediate priority is to convince more physicians, pharmacists, and patients that generic drugs are bioequivalent to branded products, although this may take time. Meanwhile, much could be achieved by requiring generic prescribing and substitution where such policies are not yet in place.

Given the mixed progress so far, it is critical to understand why previous initiatives failed. Much can be learned from policy analyses, such as the one in this paper. These typically highlight the role played by special‐interest groups in obstructing reform.

Finally, it is important to be realistic about what can be achieved. Despite some widely publicized examples of profiteering, discussed earlier, most of the growth in drug spending will continue to be driven by new medicines. For some treatments, like certain cancer immunotherapies, the complex manufacturing process means that the scope for off‐patent products is still limited. Yet, there are opportunities for significant cost savings from generics in many countries and, even where there are historically strong generic markets, like the United States, regulators, policymakers, and payers can do more to ensure timely generic drug availability.

## Appendix 1 Sample of Medicines

List of the 200 Most‐Prescribed Off‐Patent Active Ingredients in the European Union in 2013 (Anatomical Main Group in Parentheses)^a,b^   

Acetylcysteine (R/S/V)

Acetylsalicylic acid (A/B/N)

**Aciclovir (D/J/S)**

**Alendronic acid (M)**

**Alfuzosin (G)**

**Allopurinol (M)**

Alprazolam (N)

Alprostadil (C/G)

Amiodarone (C)

Amisulpride (N)

Amitriptyline (N)

**Amlodipine (C)**

Amlodipine/perindopril (C)

Amoxicillin (J)

**Amoxicillin/clavulanic acid (J)**

**Anastrozole (L)**

Apomorphine (G/N)

**Atenolol (C)**

**Atorvastatin (C)**

Azathioprine (L)

**Azithromycin (J/S)**

Beclometasone (A/D/R)

Betahistine (N)

Betamethasone (A/C/D/H/R/S)

**Bicalutamide (L)**

**Bisoprolol (C)**

Bisoprolol/ hydrochlorothiazide (C)

Bromazepam (N)

**Budesonide (A/D/R)**

Buprenorphine (N)

**Candesartan cilexetil (C)**

Candesartan cilexetil/hydrochlorothiazide (C)

Carbamazepine (N)

Carbidopa/levodopa (N)

**Carvedilol (C)**

Cefpodoxime proxetil (J)

Ceftriaxone (J)

Cefuroxime axetil (J/S)

Cetirizine (R)

Ciclosporin (L/S)

**Ciprofloxacin (J/S)**

**Citalopram (N)**

Clarithromycin (J)

**Clindamycin (D/G/J)**

**Clopidogrel (B)**

Clozapine (N)

Codeine (N/R)

Codeine/paracetamol (N)

Cyproterone ethinylestradiol (G)

Desloratadine (R)

Desmopressin (H)

Desogestrel/ethinylestradiol (G)

**Dexamethasone (A/C/D/H/R/S)**

Diazepam (N)

**Diclofenac (D/M/S)**

Dienogest/ethinylestradiol (G)

Diltiazem (C)

Docetaxel (L)

Domperidone (A)

Donepezil (N)

Doxazosin (C)

**Doxycycline (A/J)**

Drospirenone/ethinylestradiol (G)

Ebastine (R)

**Enalapril (C)**

**Enalapril/hydrochlorothiazide (C)**

**Erythromycin (D/J/S)**

Escitalopram (N)

**Esomeprazole (A)**

**Estradiol (G)**

Estradiol/norethisterone (G)

Ethinylestradiol/gestodene (G)

Ethinylestradiol/levonorgestrel (G)

Felodipine (C)

Fenofibrate (C)

**Fentanyl (N)**

**Finasteride (D/G)**

Flucloxacillin (J)

**Fluconazole (D/J)**

**Fluoxetine (N)**

Fluticasone/salmeterol (R)

Fluvastatin (C)

Formoterol (R)

**Furosemide (C)**

**Gabapentin (N)**

Galantamine (N)

Gliclazide (A)

**Glimepiride (A)**

Hyaluronic acid (D/M/R/S)

Hydrochlorothiazide (C)

**Hydrochlorothiazide/lisinopril (C)**

**Hydrochlorothiazide/losartan (C)**

Hydrochlorothiazide/ ramipril (C)

**Hydrochlorothiazide/valsartan (C)**

**Hydrocortisone (A/C/D/H/S)**

Hydromorphone (N)

**Ibandronic acid (M)**

**Ibuprofen (C/G/M)**

Indapamide (C)

Indapamide/perindopril (C)

Iodine/levothyroxine sodium (H)

Ipratropium bromide (R)

**Irbesartan (C)**

Irinotecan (L)

Isosorbide mononitrate (C)

Isotretinoin (D)

Ketoprofen (M)

**Lamotrigine (N)**

**Lansoprazole (A)**

**Latanoprost (S)**

Leflunomide (L)

Lercanidipine (C)

**Letrozole (L)**

Leuprorelin (L)

**Levetiracetam (N)**

Levocetirizine (R)

Levofloxacin (J/S)

**Levothyroxine sodium (H)**

Lidocaine (C/D/N/R/S)

**Lisinopril (C)**

**Lorazepam (N)**

Lormetazepam (N)

**Losartan (C)**

Memantine (N)

Mesalazine (A)

Metamizole sodium (N)

**Metformin (A)**

Methadone (N)

**Methotrexate (L)**

Methylphenidate (N)

Metoprolol (C)

**Metronidazole (A/D/G/J/P)**

**Mirtazapine (N)**

Molsidomine (C)

**Montelukast (R)**

Morphine (N)

Moxonidine (C)

Naloxone/tilidine (N/V)

**Naproxen (G/M)**

Nebivolol (C)

Nifedipine (C)

**Nitroglycerin (C)**

Ofloxacin (J/S)

**Olanzapine (N)**

**Omeprazole (A)**

**Ondansetron (A)**

Oxaliplatin (L)

Oxycodone (N)

Paclitaxel (L)

**Pantoprazole (A)**

Paracetamol (N)

Paracetamol/tramadol (N)

**Paroxetine (N)**

Penicillin (J/S)

Perindopril (C)

Phenytoin (N)

Piracetam (N)

Pramipexole (N)

**Pravastatin (C)**

**Prednisolone (A/C/D/H/R/S)**

Prednisone (A/H)

**Progesterone (G)**

Propranolol (C)

Quetiapine (N)

Rabeprazole (A)

**Ramipril (C)**

Ranitidine (A)

Repaglinide (A)

Rilmenidine (C)

**Risedronic acid (M)**

**Risperidone (N)**

Ropinirole (N)

Rosuvastatin (C)

**Salbutamol (R)**

**Sertraline (N)**

Sildenafil (G)

**Simvastatin (C)**

**Spironolactone (C)**

Sumatriptan (N)

Tamsulosin (G)

Temazepam (N)

Temozolomide (L)

**Terbinafine (D)**

**Testosterone (G)**

**Timolol (C/S)**

Tolterodine (G)

**Topiramate (N)**

Torasemide (C)

**Tramadol (N)**

Trazodone (N)

Trimebutine (A)

Trimetazidine (C)

**Valaciclovir (J)**

Valproic acid (N)

**Valsartan (C)**

**Venlafaxine (N)**

Verapamil (C)

Warfarin (B)

Zolpidem (N)

Zopiclone (N)

^a^The data included over‐the‐counter products, such as ibuprofen and paracetamol, if these were prescribed by a licensed health care practitioner. The active ingredients listed in bold were available in at least one strength‐form combination in each country. These 80 ingredients comprised the common sample analyzed in this paper.

^b^Reproduced from IMS Health 2013 (Pricing Insights database); anatomical main groups from the WHOCC ATC/DDD Index (2015).

## Appendix 2 Description of IMS Health Data

### Prices

IMS Health collects data on pack prices of medicines in European countries from government price lists, wholesaler invoices, and other validated sources. The company collects data at different levels of the distribution chain, based on data availability. IMS Health regularly—usually quarterly—audits price levels to obtain up‐to‐date price and volume data for each country. The company has internal quality assurance procedures.

When data are unavailable at a level of the distribution chain, IMS Health adopts the same approach taken by national health ministries, or the relevant authorities, to calculate ex‐manufacturer and/or retail prices. In Spain, for example, IMS Health only collects data on retail prices, exclusive of value‐added taxes, and then calculates ex‐manufacturer prices based on official mark‐ups regulated by the government (Table A).

**Table A:** Price Buildup of Medicines in Spain (2013)^a^

Ex‐manufacturer Price (per Pack)	Corresponding Wholesale Mark‐up
€0.00‐€91.63	7.6% of the wholesale price
€91.64 +	€7.54 (flat fee)
**Ex‐manufacturer Price (per Pack)**	**Corresponding Retail Mark‐up**
€0.00‐€91.63	27.9% of the retail price (excluding VAT)
€91.64‐€200.00	€38.37 (flat fee)
€200.01‐€500.00	€43.37 (flat fee)
€500.01 +	€48.37 (flat fee)

VAT, value‐added tax.

^a^Reproduced from IMS Health (2013).

In some countries, IMS Health collects data on wholesale prices (ie, prices charged by wholesalers to pharmacies), which they use to calculate ex‐manufacturer and retail prices. For countries where distribution margins are unregulated, IMS Health estimates average margins, which can vary by product group.

Refer to IMS Health documentation for more information about data sources in each country.

### Sales

IMS Health uses price and volume data to report aggregate sales, since a common denominator (eg, doses) is needed to compare prices across drug forms and strengths.

IMS Health calculates total sales of a product by multiplying the pack price by the number of packs sold (Table B). IMS Health relies on the latest price in a quarter. The company excludes value‐added taxes to ensure comparability across countries. The sales figures do not reflect discounts, rebates, clawbacks, and other forms of confidential price reductions.

**Table B:** Example Calculations for One Quarter^a^

Product	Country	Retail Price per Pack	# of Packs Sold	Sales Calculation	Total Retail Sales
A	Italy	€12.67	12,750	12,750 · €12.67	€161,542.50
B	Sweden	15.50 kr	5,000	5,000 · 15.50 kr	77,500.00 kr
C	United Kingdom	£8.23	7,934	7,934 · £8.23	£65,296.82

^a^Reproduced from IMS Health (2013).
